# Overutilization of ambulatory medical care in the elderly German population? – An empirical study based on national insurance claims data and a review of foreign studies

**DOI:** 10.1186/s12913-016-1357-y

**Published:** 2016-04-14

**Authors:** Hendrik van den Bussche, Hanna Kaduszkiewicz, Ingmar Schäfer, Daniela Koller, Heike Hansen, Martin Scherer, Gerhard Schön

**Affiliations:** Institute of Primary Medical Care, University Medical Center Hamburg-Eppendorf, Martinistrasse 52, 20246 Hamburg, Germany; Institute of General Practice, Christian-Albrechts-University Kiel, Kiel, Germany; Department of Health Services Management, Ludwig-Maximilians-University Munich, Munich, Germany; Institute of Medical Biometry and Epidemiology, University Medical Center Hamburg Eppendorf, Hamburg, Germany

**Keywords:** Primary care, Health services utilization, Frequent attenders, High utilizers, Multimorbidity, Elderly population

## Abstract

**Background:**

By definition, high utilizers receive a large proportion of medical services and produce relatively high costs. The authors report the results of a study on the utilization of ambulatory medical care by the elderly population in Germany in comparison to other OECD countries. Evidence points to an excessive utilization in Germany. It is important to document these utilization figures and compare them to those in other countries since the healtcare system in Germany stopped recording ambulatory healthcare utilization figures in 2008.

**Methods:**

The study is based on the claims data of all insurants aged ≥ 65 of a statutory health insurance company in Germany (*n* = 123,224). Utilization was analyzed by the number of contacts with physicians in ambulatory medical care and by the number of different practices contacted over one year. Criteria for frequent attendance were ≥ 50 contacts with practices or contacts with ≥ 10 different practices or ≥ 3 practices of the same discipline per year. Descriptive statistical analysis and logistic regression were applied. Morbidity was analyzed by prevalence and relative risk for frequent attendance for 46 chronic diseases.

**Results:**

Nineteen percent of the elderly were identified as high utilizers, corresponding to approximately 3.5 million elderly people in Germany. Two main types were identified. One type has many contacts with practices, belongs to the oldest age group, suffers from severe somatic diseases and multimorbidity, and/or is dependent on long-term care. The other type contacts large numbers of practices, consists of younger elderly who often suffer from psychiatric and/or psychosomatic complaints, and is less frequently multimorbid and/or nursing care dependent.

**Conclusion:**

We found a very high rate of frequent attendance among the German elderly, which is unique among the OECD countries. Further research should clarify its reasons and if this degree of utilization is beneficial for elderly people.

## Background

By definition, high utilizers (synonymous with “high users” and “frequent attenders”) receive a large proportion of medical services and therefore produce relatively high costs [1, 2]. In an earlier study we found that the elderly statutorily insured population in Germany has an average of 27.9 practice contacts per year (SD 23.6; median 23), spread over 4.8 physician practices (SD 3.3; median 4) [3]. This number of practice contacts is about two thirds higher than the 16 to 18 contacts found for the insured population as a whole during the first decade of the 21^st^ century [4]. A specific look at the 60 % of the elderly population that is multimorbid – defined as suffering from ≥ 3 chronic diseases from a list of 46 – revealed that this sample of patients had 36.3 contacts/year (SD 24.5; median 31), almost 30 % more than the average, whereas the number of different practices contacted by this sample (5.7) was only slightly higher (SD 3.3; median 5) [3].

In the majority of studies from other countries, high utilization is associated with chronic diseases and multimorbidity [5–7]. In several studies, associations were found between high utilization and psychological stress, psychiatric syndromes, and medically unexplained symptoms (“MUS”) [8–11]. On the other hand, studies on the influence of somatic diseases on frequent attendance are rare. Several studies also report a high sensitivity towards health and disease in high utilizers, which may lead to complaints and increased health fears. This is especially true in cases where the psycho-social explanations given by patients concerning the source of their illness are not accepted by their physicians [12, 13]. In several studies it was also noted that patients were critical of what they perceived as excessive somatic diagnostic examinations and an ineffectiveness of their physicians’ treatments [14–16].

Our previous study, mentioned above [3], showed an almost linear association between the number of chronic conditions and the number of physician contacts. However, high standard deviations were found for each number of chronic conditions. This result posed the question of the reasons for this degree of variance, leading to an in-depth examination of the high utilizers (abbrev: HU) in every subsample. Therefore, this study tries to answer the following questions: a) Which proportion of the elderly population are high utilizers of ambulatory care in Germany? b) Can different types of high utilization be identified? c) Which socio-demographic factors and morbidity patterns are associated with high utilization? d) To which extent are data from other countries comparable to the German data?

### The German ambulatory healthcare system

In order to interpret data on healthcare utilization in Germany and understand the conclusions of this study, a brief introduction to the structure of the German healthcare system is provided here [17]. Health insurance is mandatory for the entire German population today, either within a “Bismarckian” statutory health insurance scheme or - for about 10 % of the population - a private one. The number of healthcare resources – physicians, nurses, physiotherapists etc. – per person is among the highest in Europe. This is also the case for inpatient hospital care [18, 19]. Ambulatory care and inpatient hospital care are separate sectors with separate budgets within the healthcare system. As a result, only few hospital physicians work simultaneously in ambulatory care and vice versa. A dense supply of both primary and specialist services exists in ambulatory care, mainly provided by physicians in solo or small group practices. General practitioners (GPs) also care for patients in nursing homes. The group of primary care physicians (PCPs) in ambulatory care consists of all GPs and those general internists opting to work as a PCP at the moment of opening their practice, which is the case for approximately 60 % of the latter. Remuneration is based on complex algorithms consisting of capitation fees (especially for PCPs) combined with fees-for-service (especially for specialists), limitations for performance maxima per quarter and extrabudgetary incentives. The average physician to population ratio is currently 1:1,433 for PCPs and 1:1,218 for specialists [20]. In metropolitan areas, the density of specialists in the ambulatory sector is already much higher than that of PCPs.

All physicians in practices work as entrepreneurs in a market characterized by free access to all medical disciplines without compulsory gatekeeping, although most referrals are based on a document of another physician, usually the GP, especially among the elderly. Starting in 2004, visits to specialists without a referral from a GP were to be curbed by the introduction of a co-payment of €10 in each quarter. This measure was abolished at the end of 2012. In addition to physicians’ practices, hospital emergency and outpatient units also provide a growing number of ambulatory services.

## Methods

The study is based on the health insurance claims data of a primary care population consisting of all members aged 65 and over (*n* = 123,224) from a statutory health insurance company operating nationwide in Germany, the Gmünder ErsatzKasse (GEK). The GEK insured 1.7 million people in 2004, a figure corresponding to approximately 2.4 % of the statutorily insured German population. The year 2004 was chosen for two reasons: a) to allow comparisons with our previous studies on multimorbidity and utilization of ambulatory care [3, 21], and b) because precise data on the utilization frequency in ambulatory care were only published until 2007; since 2008, contacts with physicians are no longer recorded and have been replaced by case counts per quarter, implying one count per quarter regardless of the real number of contacts during the quarter. Therefore, claims data based utilization research in Germany can only reply on older data and draw estimates from them for the current situation (see discussion chapter). As in the years before 2008, there are good reasons to presume a further growth of utilization, e. g., because of the increasing average age of the population.

The claims data were provided by the GEK in a pseudonymous form. Insurants were included in this study when they were aged ≥ 65 years, were insured for the whole year of the investigation, and had at least one contact with one ambulatory care physician during that year (96 % of the total sample). The 4 % non-users were excluded because they do not appear in the dataset of the year under investigation.

Utilization of ambulatory medical services was ascertained through the number of contacts per year in all contacted practices, and the number of different physician practices contacted. The term “contact” includes all kinds of utilization, such as personal consultation in the office and/or during home and nursing home visits, regardless of their length, as well as some contacts with the practice staff (e.g., for prescription renewals) and/or telephone contacts with the physician, the latter being infrequent in Germany. Contacts deserved a presence of the patient in the practice, or with the physician. Appointments by phone and/or administrative work including contacts with other physicians concerning a specific patient were not counted as contacts. Several combinations and gradations within an encounter are possible. Therefore we preferred the term “contact” over “visit” or “consultation”, and we use the term “practice” to cover the contacts with all practice members.

**Fig. 1:**
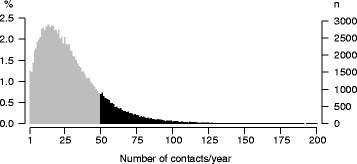
Distribution of practice contacts/year in the population under study (*n* = 123.224) (number of persons with ≥ 50 contacts/year in black)

Figure 1 shows the distribution of the number of practice contacts per year in the total sample (except for those without any contact in the year of observation), corresponding to a mean of 28 contacts per year (median 23). Since there is no generally accepted definition of high utilization [22, 23], we set the lower cutoff for high utilization deductively at 50 contacts per year, almost one per week. This criterion corresponds to 14.2 % of the sample (*n* = 17,552).

**Fig. 2:**
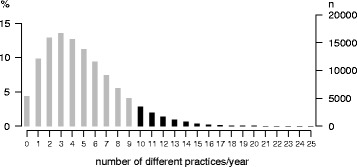
Distribution of the number of different practices contacted by the study population (*n* = 123.224) (number of persons with ≥ 10 contacted practices/year in black; cut at 25, maximum: 38 practices)

On average, the elderly contacted 5 different practices per year (median 4) [3]. As a second criterion for high utilization, we examined the subsample that contacted ≥10 different practices per year. This criterion corresponds to 8.9 % of the sample (*n* = 10,958) (Fig. 2).

Usually, contacts with N different practices contacted per year means contacts with 1 GP and N-1 specialists (except for type C defined below by contacts to more than 1 specialist of a particular discipline). As a subgroup of the insurants that contacted several practices of the same medical specialization during the year of observation, particular consideration was given to those who contacted ≥ 3 practices of the same specialization (with the exception of primary care or internal medicine). This criterion corresponds to 5.1 % of the sample (*n* = 6,224). The criterion of ≥ 3 practices of the same specialization was chosen because a cutoff of two contacts would have defined all those seeking a second opinion or visiting a holiday replacement physician as high utilizers.

As a result, high utilizers in this study are defined by one or more of the following characteristics:

- Insured persons with ≥ 50 contacts with ambulatory care physician practices within a year (HU-type A),
- Insured persons with one or more contacts to ≥ 10 different physician practices within a year (HU-type B),
- Insured persons with one or more contacts to ≥ 3 different physician practices of the same medical specialization (except for general practice or internal medicine) within a year (HU-type C).

Since an insured person can belong to more than one of these three high utilizer types, seven subtypes can be identified (A--, B--, C--, AB-, ABC, AC-, and BC-). These seven subtypes are free of any overlap.

In order to analyze multimorbidity, we used a list of 46 ICD-10 diagnoses or diagnosis groups, which covered all chronic conditions with a prevalence ≥1 % in the age group ≥ 65 years in the GEK dataset. The process of creating this list is described in detail in a previous publication [21]. A person was defined as chronically ill if he or she had at least one chronic condition from this list over ≥ 3 quarters of the observation year, and defined as multimorbid if he or she had ≥ 3 chronic conditions from this list over ≥ 3 quarters of the observation year. Nursing care dependency was assumed when services of a statutory nursing care insurance scheme, a parallel agency to the statutory health services insurance, were received for at least six continuous months during the observation year. This criterion is used as a proxy for disability, but it should be understood that this underestimates the prevalence of disability because disability with no or little impact on activities of daily life (“ADL”) does usually not lead to the receipt of benefits from nursing care insurance.

The main statistical analyses consist of multidimensional frequency tables with the corresponding percentages. Arithmetic means and medians, standard deviations, and confidence intervals were calculated for all continuous variables. A linear regression model was developed in order to determine the independent effect of patient characteristics on physician utilization (age, gender, nursing care dependency [yes/no], and the logarithm of the number of chronic conditions). Because of the interaction between age and gender, four combinations were analyzed with the subsample of men aged 65 to 74 years as a reference. The odds ratios express the relative chance of a variable to predict a person’s relationship to a HU-subgroup under the control of all other variables.

To detect the prevalence of single chronic conditions in high utilizers, relative risks were calculated. The relative risk expresses to which extent a variable (in this case: a chronic condition) is associated with a specific risk (in this case: high utilization). Relative risks are comparable to odds ratios, but they are less dependent on prevalence differences between diseases than odds ratios [24, 25].

As for the literature review, we searched PubMed and Medline for articles about utilization in ambulatory medical care. We used the following terms (combining them with “and” and “or”): ambulatory care/outpatient care/primary care, doctor/physician – patient contact, utilization/use/frequency, and elderly/multimorbidity. We limited our results to papers written in English, German, French and Dutch, and to papers published after 1990. We also searched the www for statistical reports of governments, insurers and physician organizations. We included all studies and reports pertaining to the topic.

All analyses were performed with the SAS statistical software (version 9.2) and SPSS 16. Figures were created using R (version 2.15.1) and Windows Microsoft Excel 2003.

The study was approved by the Ethics Committee of the Medical Association of Hamburg (approval no. PV3057).

## Results

### Study population

The total study population consists of 123,224 patients, 57.8 % of which were male. The mean age was 70 years (70 for men and 71 for women). 60 % of the total population was multimorbid according to the criterion of ≥ 3 chronic conditions (average number of chronic conditions 3.6 [3.9 for females and 3.5 for males]). The percentage of nursing care dependent persons was 13.4 (16.0 for females and 11.4 for men).

In the HU-sample, the mean age was 73.0 years (median (Md) 72) compared to 71.7 years (Md 70) in the non-high utilizer sample (NHU-sample). The average number of chronic conditions was 5.8 (Md 5) in the HU-sample compared to 3.1 (Md 3.0) in the NHU-sample; as a result, the percentage of multimorbid persons was 84.7 in the HU-sample compared to 52.8 in the NHU-sample. 11.7 % of the high utilizers were nursing care dependent, compared to 4.1 among the non-high utilizers.

### Prevalence and typology of high utilization

Nineteen percent of the insured elderly were high utilizers according to the study criteria. HU-type A was found in 14.2 % of the elderly population and 74.4 % of the HU-sample, respectively; HU-type B in 8.9 % of the elderly population and 46.4 % of the high utilizers. HU-type C was the least frequent (5.1 % of the elderly population and 26.4 % of the HU-sample). Thus, more than a quarter of high utilizers contacted three and more physicians of the same discipline (general practice and internal medicine excluded) per year. The addition of the three types of high utilizers amounts to 147 %, which implies that more than one high utilization criterion applies to a substantial percentage of the HU-sample.

High utilizers had an average of 61.9 practice contacts per year (A: 70.7, B 62.9, and type C 57.7 contacts), three times more than non-high utilizers. The average number of contacted practices in the HU-sample was 9.0 (A: 8.8, B: 12.0, C 10.5), compared to 3.8 in the NHU-sample. Differences between the three types were relatively small with regard to age and gender, and this also applies to these respective differences between the HU- and the NHU-sample. Compared with type A, persons belonging to types B and C were somewhat younger, less multimorbid, and less dependent on nursing care.

At the level of the subtypes, combinations without an A component (B--, BC- and C--) had only half as many contacts as those with an A component. HU-type A-- dominates the distribution with 41.8 % prevalence. Subtype ABC, corresponding to 10 % of the high utilizers, shows the highest average number of contacts (83/year = 1.6/week) and contacted practices (13.7) (see Table 1).

**Table 1:** Characteristics of high user types and subtypes

	NHU	HU	HU-types			HU-subtypes						
			A	B	C	A - -	AB -	ABC	AC-	B--	BC-	C--
Sample size (%)	99,634 (80.9)	23,590 (19.1)	17,552 (14.2)	10,958 (8.9)	6,224 (5.1)	9,859 (8.0)	4,555 (3.7)	2,460 (2.0)	678 (0.6)	2,952 (2.4)	991 (0.8)	2,095 (1.7)
Percentage of high users (%)	0	100	74.4	46.4	26.4	41.8	19.3	10.4	2.9	12.5	4.2	8.9
Mean contacts with practices (SD)	19.9 (12.8)	61.9 (28.1)	70.7 (27.0)	62.9 (30.4)	57.7 (37.5)	66.3 (22.2)	72.2 (23.2)	83.0 (39.5)	76.2 (38.9)	39.2 (7.1)	39.3 (6.8)	30.9 (9.9)
Mean contacted practices (SD)	3.8 (2.3)	9.0 (3.5)	8.8 (3.8)	12.0 (2.3)	10.5 (3.7)	6.3 (2.1)	11.9 (1.9)	13.7 (3.2)	8.0 (1.1)	10.8 (1.2)	11.5 (1.6)	7.1 (1.4)
Multimorbid patients (%)	52,559 (52.8)	19,989 (84.7)	15,664 (89.2)	9,333 (85.2)	4,917 (79.0)	8,665 (87.9)	4,180 (91.8)	2,220 (90.2)	599 (88.3)	2,227 (75.4)	706 (71.2)	1,392 (66.4)
Females (%)	41,297 (41.4)	10,920 (46.3)	8,084 (46.1)	5,184 (47.3)	2,614 (42.0)	4,641 (47.1)	2,153 (47.3)	1,047 (42.6)	243 (35.8)	1,512 (51.2)	472 (47.6)	852 (40.7)
Mean age [SD] (Md)	71.7 [6.1] (70)	73.0 [6.4] (72)	73.7 [6.6] (73)	71.7 [5.6] (70)	71.8 [5.6] (70)	74.7 [7.0] (74)	72.4 [5.8] (71)	72.1 [5.6] (71)	73.2 [6.1] (72)	70.7 [5.3] (69)	70.5 [4.9] (69)	71.6 [5.7] (70)
Age ≥ 75 years (%)	28,603 (28.7)	8,849 (37.5)	7,375 (42.0)	3,264 (29.8)	1,899 (30.5)	4,710 (47.8)	1,562 (34.3)	830 (33.7)	273 (40.3)	678 (23.0)	194 (19.6)	602 (28.7)
Nursing care dependents (%)	4,046 (4.1)	2,749 (11.7)	2,630 (15.0)	477 (4.4)	333 (5.4)	2,111 (21.4)	261 (5.7)	155 (6.3)	103 (15.2)	44 (1.5)	17 (1.7)	58 (2.8)
Mean chronic conditions [SD] (Md)	3.1 [2.9] (3)	5.8 [3.3] (5)	6.2 [3.2] (6)	5.9 [3.3] (5)	5.3 [3.3] (5)	6.0 [3.2] (6)	6.7 [3.3] (6)	6.5 [3.3] (6)	6.0 [3.1] (6)	4.6 [2.9] (4)	4.4 [2.8] (4)	4.0 [2.8] (4)

*NHU* non high user, *HU* high user, *SD* standard deviation, *Md* median

To test the associations described above, logistic regressions were calculated with age and gender in four different combinations, using males aged < 75 years as a reference group (see Methods section), nursing care dependency (yes/no), and the logarithmic number of chronic diseases. Except for one, all odds ratios (OR) presented in Table 2 are statistically significant (*p* < 0.01). Table 2 shows that the relative chance of belonging to type A was significantly higher in the oldest age group for both genders but significantly lower for the younger elderly females compared to the younger elderly males. Inversely, the chance of belonging to type B was highest in the younger female group but much smaller in both oldest age groups. Furthermore, the chance of belonging to type C was highest in the younger male group.

**Table 2:** Significant (*p* < 0.01) odds ratios with their 95 % confidence intervals (in parentheses) for the affiliation to a high user type

	High users total	Type A	Type B	Type C
Males aged ≥ 75	1.19 (1.14–1.25)	1.38 (1.26–1.51)	0.79 (0.73–0.85)	0.88 (0.81–0.95)
Females aged 65–74	1.27 (1.22–1.32)	0.83 (0.77–0.89)	1.38 (1.29–1.47)	0.88 (0.82–0.95)
Females aged ≥ 75	n.s.	1.55 (1.40–1.72)	0.65 (0.60–0.71)	0.60 (0.55–0.66)
Nursing care dependency	2.49 (2.36–2.62)	7.00 (5.93–8.34)	0.28 (0.26–0.31)	0.43 (0.38–0.48)
Number of chronic conditions	1.28 (1.28–1.29)	1.23 (1.22–1.24)	1.04 (1.03–1.05)	0.94 (0.93–0.95)

Reference for age and sex = males aged 65–74; *n.s.* not significant

Types B and C were only marginally related to multimorbidity, as every additional chronic disease increases the chance of belonging to type B by only 4 % and decreases the chance of belonging to type C by 6 %. Being dependent on nursing care even reduces the chance by 72 % for type B and 57 % for type C. On the other hand, type A was closely associated with multimorbidity and nursing care dependency, as the chance of belonging to this type grew by 23 % for every additional chronic disease, whereas being nursing care dependent increased this chance by a factor of 7. HU-type C was almost independent of the number of chronic conditions and of nursing care dependency in particular, as the latter lowered the chance of belonging to C by more than 50 %. In sum, HU-types B and C differed substantially from HU-type A with regard to their relation to age, sex, multimorbidity, and nursing care dependency.

### Which chronic diseases are associated with high utilization?

**Fig. 3:**
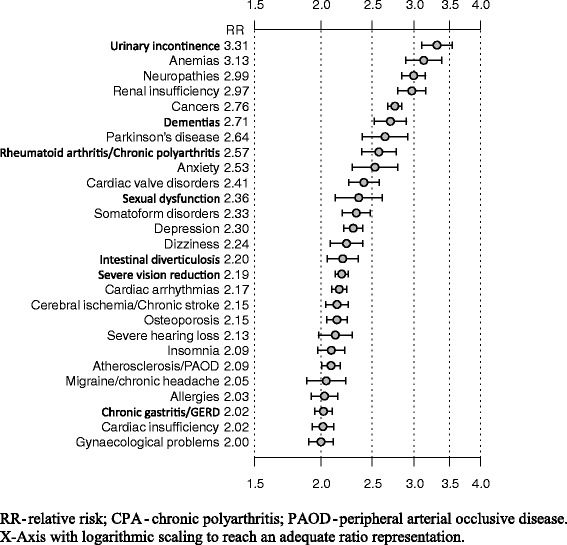
Relative risk for high utilization with 95 % confidence intervals for those chronic diseases with a relative risk ≥ 2

As Fig. 3 shows, the relative risks for becoming a high utilizer due to single diagnoses above a doubled risk spanned from 3.3 for urinary incontinence to 2.02 for cardiac insufficiency. Thus, patients with urinary incontinence have a 3.3 times higher chance of being a high utilizer than patients without urinary incontinence. The lowest risk for becoming a high utilizer showed hyperlipidemia (1.3).

Additionally, Table 3 shows the prevalences and relative risks for the 10 diagnoses with the highest relative risk for each of the three high utilization types. For example, the relative risk of belonging to HU-type A is associated with several high impact somatic diseases (e.g., urinary incontinence, anemia, renal insufficiency, and neuropathy) as the top 4 of the list (RR for all four ≥ 3.4). Contacting more than 10 different practices (type B) and/or contacting ≥ 3 practices of the same discipline was primarily associated with psychological disorders (anxiety, somatoform disorders) and/or diagnoses often assumed to be linked to psychiatric conditions (e.g., sexual disorders, migraine/chronic headache, and selected gynecological complaints).

**Table 3:** Prevalences and relative risks (with their 95 % confidence intervals) of the 10 diagnoses with the highest risk for high use according to high use types

RR-rank	High use total (*n* = 23,590)			High use type A (*n* = 17,552)			High use type B (*n* = 10,958)			High use type C (*n* = 6,224)		
	Diagnosis	n (prev)	RR (95 % CI)	Diagnosis	n (prev)	RR (95 % CI)	Diagnosis	n (prev)	RR (95 % CI)	Diagnosis	n (prev)	RR (95 % CI)
1	Urinary incontinence	1488 (6.3)	3.3 (3.1–3.5)	Urinary incontinence	1317 (7.5)	3.9 (3.7–4.2)	Neuropathy	1177 (10.7)	3.3 (3.1–3.5)	Cancer	1860 (29.9)	3.4 (3.3–3.6)
2	Anemia	1059 (4.5)	3.1 (2.9–3.4)	Anemia	932 (5.3)	3.7 (3.4–4.0)	Cancer	2906 (26.5)	3.0 (2.9–3.2)	Renal insufficiency	511 (8.2)	3.4 (3.1–3.8)
3	Neuropathy	2327 (9.9)	3.0 (2.8–3.2)	Renal insufficiency	1466 (8.4)	3.5 (3.3–3.7)	Anxiety disorder	345 (3.1)	3.0 (2.7–3.4)	Anemia	302 (4.9)	3.4 (3.0–3.8)
4	Renal insufficiency	1677 (7.1)	3.0 (2.8–3.2)	Neuropathy	1978 (11.3)	3.4 (3.2–3.8)	Anemia	469 (4.3)	3.0 (2.7–3.3)	Sexual disorder	186 (3.0)	2.9 (2.5–3.4)
5	Cancer	5660 (24.0)	2.8 (2.7–2.8)	Dementias	1175 (6.7)	3.4 (3.2–3.7)	Somatoform disorder	880 (8.0)	2.9 (2.7–3.1)	Neuropathy	529 (8.5)	2.6 (2.4–2.8)
6	Dementias	1252 (5.3)	2.7 (2.5–2.9)	Parkinson’s disease	556 (3.2)	3.2 (2.9–3.5)	Sexual disorder	312 (2.8)	2.8 (2.5–3.2)	Rheumatism/CPA	267 (4.3)	2.4 (2.2–2.8)
7	Parkinson’s disease	619 (2.6)	2.6 (2.4–2.9)	Rheumatism/CPA	890 (5.1)	2.9 (2.7–3.1)	Rheumatism/CPA	524 (4.8)	2.7 (2.5–3.0)	Urinary incontinence	270 (4.3)	2.3 (2.0–2.6)
8	Rheumatism/CPA	1065 (4.5)	2.6 (2.4–2.8)	Cancer	4402 (25.1)	2.9 (2.8–3.0)	Urinary incontinence	547 (5.0)	2.6 (2.4–2.9)	Severe vision loss	1570 (25.2)	2.3 (2.2–2.4)
9	Anxiety disorder	623 (2.6)	2.5 (2.3–2.8)	Anxiety disorder	522 (3.0)	2.8 (2.6–3.2)	Bowel diver-ticulosis	685 (6.3)	2.6 (2.4–2.8)	Gynecological complaints	554 (8.9)	2.2 (2.0–2.4)
10	Heart valve disorders	1329 (5.6)	2.4 (2.3–2.6)	Heart valve disorders	1126 (6.4)	2.7 (2.6–2.9)	Migraine/chron. headache	449 (4.1)	2.6 (2.3–2.9)	Anxiety disorder	137 (2.2)	2.1 (1.8–2.5)

*n* absolute number, *prev* prevalence, *RR* relative risk, *CI* confidence interval, *CPA* chronic polyarthritis

Example: 1488 patients suffering from urinary incontinence are found in the HU-sample, a figure corresponding to a prevalence of 6.3 %; a patient suffering from urinary incontinence has a risk to belong to HU-type A which is 3.9 points higher than the one to belong to the NHU-subsample. The confidence intervals point to statistical significance

## Discussion

### Epidemiological summary

According to the criteria used in this study, it is estimated that, depending on the study population (see strengths and weaknesses section) some 20 % of the ≥ 65 year-old, statutorily insured German population are to be considered high utilizers of outpatient care. This figure corresponds to approximately 3.5 million persons covered by statutory health insurance. The frequent attenders in this study had on average 62 contacts with an ambulatory care practice (1.2 contacts per week) spread over 9 different physicians, usually 1 GP and 8 specialists, per year. Two main HU-types were found. The first type (HU-type A) is characterized by the large average number of practice contacts (71/year = 1.4 contacts/week), found predominantly among the oldest age group (42 % ≥ 75 years), in patients with a high percentage of multimorbidity (89 %), and relatively often in need for nursing care (15 % as compared to 11 % in the general population in this age group). This type corresponds to the traditional public conception of a high utilizer as a very old person suffering from multiple burdening diseases that require continuous medical and nursing services. The other HU-types - a combination of HU-types B and C - were comparably prevalent but consisted of a significantly younger age group (30 % ≥ 75 years) that was somewhat less frequently multimorbid (83 %), and which particularly less frequently received nursing care (5 %). These HU-types consulted many different physicians (11 - 12 per year), often from the same specialization (36 %). These types of high utilization are frequently called “doctor (s)hopping” in the literature [26, 27]. Remarkably, gender differences were not found.

The criteria for this typology were set deductively on the basis of the empirical distribution of the data. Therefore it should be kept in mind that using stricter or more tolerant criteria for frequent attendance would have lead to different results. For example, using ≥ 8 instead of ≥ 10 different practices as a cutoff for HU-type B would have raised the percentage of B from 9 to 12 % of the elderly population; inversely, a cutoff of ≥ 2 instead ≥ 3 practices of the same discipline would have raised the percentage of C-type high utilizers from 5 to 24 %. In other words, a quarter of the elderly contacts ≥ 2 specialist practices of the same discipline per year.

### Morbidity patterns of high users

High utilization of statutory ambulatory care is related to specific diagnoses. All 46 ICD-10 diagnoses for chronic conditions investigated in this study showed a minimum odds ratio of 1.3 for belonging to the high utilizer sample, and 27 of 46 had a respective odds ratio ≥ 2. Also, complex associations were found between diagnoses and HU-type. On the one hand, several chronic conditions were found in the list of top 10 relative risks for all three HU-types A, B, and C, albeit at different positions in the list for each type. This is the case for urinary incontinence, cancer, anemia, neuropathy, chronic polyarthritis, and anxiety (see Table 3). In diagnoses like cancer, the most prevalent disease of the ABC-subtype, the combination of serious somatic disease, psychological stress, and the search for further treatment options could be the reason for often contacting several practices, including those of the same specialization. Neuropathy also, the top ranking disease in type B, might fall under the category of symptoms that are difficult to treat, resulting in consultations with multiple physicians. On the other hand, certain groups of chronic conditions are specific for single types of high use. Dementia and Parkinson’s disease, for example, only appear among the diseases associated with many contacts (type A) but not with consulting many specialists; in the case of dementia, contacts are centered mainly around the GP [28]. Furthermore, several syndromes with a possibly psychiatric background are found especially in HU-types B and C, such as somatoform disorders, sexual problems, anxiety, and migraine/headache. Very probably, these patients migrate through the care system and contact many different specialists because of a lack of satisfaction with the explanations or treatments offered to them. The relevance of psychiatric and psychosomatic factors for “doctor (s)hopping” has been confirmed in many international studies [13, 16, 27], as well as in German studies. In a representative study of the general population in Germany, Mewes et al. found a higher level of somatization and/or post-traumatic symptoms to predict high utilization [29]. Schneider et al. found a higher utilization rate of GP practices by patients with recorded psychiatric diagnoses compared to those with somatic diagnoses [30]. Apart from morbidity, for a patient to become a high utilizer of a particular type is probably dependent on additional factors, such as the burden of comorbidity [8], and vulnerability [31]. Furthermore, personality variables (e.g., subjective disease concepts [32]), personality traits [33], situational factors (e.g., regional service supply differences or previous experiences with the healthcare system) might play a role. Further research is needed in order to identify such factors.

### German utilization figures in international comparison

With an average of 28 contacts per year distributed over 5 physicians in the total elderly population, 36 contacts per year spread over 6 physicians in the multimorbid elderly subsample, and 62 contacts per year with 9 different physicians in the 19 % HU-subsample, Germany seems to be at the top of the rank order of ambulatory utilization in OECD countries as shown in Table 4 which summarizes the results of a review of 27 empirical studies on the extent and frequency of utilization of ambulatory care in several OECD countries, with special reference to utilization by the (multimorbid) elderly and high utilization.

**Table 4:** Studies on physicians’ work load in ambulatory medical care, length of consultations, number of contacts and contacted physicians per year, referral frequency to specialist care with special reference to high use among the (multimorbid) elderly

	Authors	Country	Sample/age	Year	Patients per week (consultation length in minutes)	Contacts per year	Contacted physi-cians per year	Referrals to specialists per year	Definition of contact In- and exclusions Definition high use
1	van den Brink-Muinen et al. [60, 61]	Seven	General practice patients age ≥ 18 (mean age: 40–48)	1997–1998	GE: 309 (7.6) BE: 149 (15.0) SWI: 126 (15.6) ES: 183 (7.8) NL: 189 (10.2) UK: 205 (9.4)	Not examined	Not examined	17.9 % (ES) −5.6 % (GE) of consultations	Encounters in practice premises, plus twice the number of home visits, plus half the number of telephone contacts; high use not examined
2	Koch et al. [40]	Eleven (seven reported here)	Survey among PCP	2009	GE: 242 (9.1) FR: 110 (22.2) IT: 171 (10.3) SWE: 53 (28.8) NL: 123 (15.0) UK: 130 (13.3) USA: 96 (22.5)	Not examined	Not examined	Not examined	“Visits”; no further specification of contact type; high use not examined
3	Starfield et al. [7]	USA	5 % Medicare sample age ≥65 according to 3 comorbidity degrees	1999	Not examined	Lowest comorbidity degree: 3.9 (2.1 PCP, 1.8 NPCP) highest comorbidity degree: 15.6 (6.6 PCP, 9.0 NPCP)	Not examined	Not examined	PCP: geriatricians included; contacts in physician practice + ED + OPD were counted highest comorbidity degree: persons with ≥ 10 chronic conditions
4	Starfield et al. [42]	USA	patients aged ≥65 in Medicare managed care	2001	Not examined	11.6 (2.7 PCP, 8.9 NPCP)	4.8 (0.8 PCP, 4.0 NPCP)	Not examined	GPs and internists included (same year hospitalized patients excluded); high use not examined
5	National Center for Health Statistics [44]	USA	National sample aged ≥65	2000	18.1 min	7 (all physicians in practice; 6.1; OPD + ED: 0,9)	Not examined	Not examined	High use not examined
6	NAMCS [62]	USA	Survey of national sample of physicians	2008–2009	Not examined	3.4 (1.9 PCP, 1.5 NPCP) patient age ≥ 65: 7,4	Not examined	Referral rate. 10.7 of visits	Visits to practices and CHCs only; telephone contacts and (nursing) home visits excluded; high use not examined
7	Barnett et al. [63]	USA	National sample aged ≥65	2009	Not examined	3.7	Not examined	All physicians: 8.6 % (PCP: 9.9 %, NPCP: 7.3 %) OPD: 16.6 %	Contacts in physician practices + OPD were counted; institutionalized patients excluded; high use not examined
8	NIVEL [37]	Netherlands	National sample aged ≥ 15 years with ≥ 1 chronic condition	2008	Not examined	2008: 9,7 (PCP: 4,6; NPCP 5,1)	Not examined	80 % referred	GPs + GP-assistants; no further specification of contact type; high use not examined
9	Cardol et al. [64]	Netherlands	Primary care patients ≥65	2000–2002	PCP: 10,2 min	Age ≥65: PCP 16.4 (age 65–74: PCP 11.6)	Not examined	Not examined	Visits + home visits + telephone + paperwork by GP + GP-assistants (telephone contacts account for 11 %); high use not examined
10	van Oostrom [38]	Netherlands	Primary care patients, age ≥65	2006–2008	Not examined	≥2 chronic conditions + age 65–74: PCP 19.6 + age ≥ 75: PCP 24.0	Not examined	mm: 36 % referred with 0,5 referrals/year	Consultations, telephone contacts (9.8 % for mm) and home visits; high use not examined
11	van den Berg [65]	Netherlands	Primary care patients; all ages	1987 & 2001	2001: 9.8 min	Not examined	Not examined	Not examined	Practice consultations only; high use not examined
12	Nie et al. [39]	Canada (Ontario)	Insured population aged ≥ 65	2005–2006	Not examined	10.3 (=6.2 PCP, 4.1 NPCP + ED); hu = 43.6 (PCP 20.7, NPCP 22.9)	Not examined	Not examined	“Office visits”; no further specification of contact type; hu-cutoff: ≥ 26 contacts (≥15 PCP visits, ≥ 11 NPCP visits, ≥ 5 ED visits) = 5,5 % of study population
13	Demers [26]	Canada (Quebec)	Insured general population	1991	Not examined	5.5 (PCP 3.6, NPCP 1.9)	3 (PCP 2, NPCP 1)	Not examined	“Encounters” not further specified; hu-cutoff: contacts with > 20 physicians (=0.06 % of patients)
14	Reid et al. [66]	Canada (Brit. Columbia)	General population ≥ 18 years	1996–1997	Not examined	hu: 50.3, nhu: 9.0	hu: 9, nhu: 2.7	Not examined	“Encounters” not further specified; ED-visits excluded; hu-cutoff: most costly 5 % of users of fee-reimbursed services
15	Broemeling et al. [67]	Canada (Brit. Columbia)	Insured general population ≥ 18 years	2000–2001	Not examined	≥1chronic condition: 11.5 (8,5 PCP + 3.0 NPCP) maximum hu: 28.1 (19.3 PCP + 8.8 NPCP)	Not examined	Not examined	“Visits”; no further specification of contact type; hu-cutoff: 5 % of total population, 11.6 % of persons with chronic conditions
16	Britt et al. [68]	Australia	Survey among GPs	2009–2010	15,3 min	Not examined	8.4 % of encounters with GP	Not examined	Consultations, home visits, nursing home visits included; high use not examined
17	Busato et al. [69]	Switzerland	Primary care sample age ≥ 40	2004	Not examined	3.0 (PCP only)	Not examined	Not examined	Specialist consultations & ED visits excluded; high use not examined
18	Bähler et al. [34]	Switzerland	Helsana Group insurants age ≥ 65	2013	Not examined	All physicians: 13.1 (mm: 15.7, nmm: 4.4) PCPs: 6.1 (mm: 7.4, nmm: 1.9) NPCP: 4.3 (mm: 5.1, nmm: 1.8)	All physicians: 2.9 (mm: 3.3, nmm: 1.5) PCP: 1.1 (mm: 1.2, nmm: 0.6) NPCP: 1.8 (mm: 2.1, nmm: 0.9)	Not examined	Consultations, home visits, OPD contacts, phone contacts (all physicians: 5.7 %) included; nursing home visits excluded mm : ≥ 2 chronic conditions; high use not examined
19	OBSAN [35]	Switzerland	Population sample age ≥ 65	2012	Not examined	8.0 (PCP: 4.2, NPCP: 3.8)	Not examined	Not examined	Visits (“Besuch einer Praxis”) included; no further specification of contact type; high use not examined
20	Neal et al. [36]	United Kingdom	Sample from 4 primary care practice	1991–1995	Not examined	10.7	Not examined	Not examined	Visits and outpatient contacts; no further specification of contact type; high use not examined
21	Salisbury et al. [70]	United Kingdom	Primary care sample age ≥ 18	2005–2008	Not examined	mm: 9.4, nmm: 3.8	Not examined	Not examined	
22	Bellón et al. [71]	Spain (Andalousia)	208 hu age ≥ 15 in one health center	2001	Not examined	21.8–22.5	Not examined	Not examined	ED/OPD-contacts excluded; no further specification of contact type hu cut-off: 14.7 for females, 13.8 for men
23	Luciano al. [72]	Spain (Catalonia)	GP sample age ≥ 65 with ≥ 3 chronic conditions	2005–2006	Not examined	23,1 (age ≥ 65: 22.4)	Not examined	Not examined	ED/OPD-contacts excluded; no further specification of contact type hu:10 % highest users = consultation frequency > 12
24	Bergh et al. [73]	Sweden	1 health center sample age ≥ 65	1997–1998	Not examined	GP-contacts: 1.2–1.4 (hu: 5, nhu: 1)	Not examined	Not examined	hu: 10 % highest users
25	Moth et al. [74]	Denmark	Primary care sample age ≥ 40	2009	(>2/3 of contacts <15 min	Not examined	Not examined	Not examined	Contacts = face-to-face, phone, email and home visits (telephone + mail-contacts 39.1 %); high use not examined
26	Drees [75]	France	PCP-population	2002	(PCP 15 min, NPCP 15–30 min)	Not examined	Not examined	Not examined	Practice consultations and home visits, no ED/OPD contacts; high use not examined
27	Health Insurance Authority [76]	Austria	“Care intensive” patients (cip); no age limit	2006–2007	Not examined	All physicians: cip: 39.6, ncip: 7 (PCP: cip: 30, ncip: 5; NPCP: cip: 9.6, ncip: 2)	Not examined	Not examined	No specification of contact type cip: numbers of contacts & services + hospital days (=7 % of population)

*Abbreviations GE* Germany, *BE* Belgium, *SWI* Switzerland, *ES* Spain, *NL* Netherlands, *UK* United Kingdom, *FR* France, *IT* Italy, *SWE* Sweden, *USA* United Staes of America, *PCP* primary care physicians, *NPCP* non-primary care physicians (specialists), *ED* emergency department, *OPD* outpatient department, *CHC* community health center, *hu* high use, *mm* multimorbid, *nmm* non-multimorbid, *cip* care intensive patients, *ncip* not care intensive patients

The top position is also found for the referral rate to secondary ambulatory care by a primary physician (93 % of the elderly at least once in a one-year period) and – inversely – for the length of contact with a physician in minutes.

Table 4 shows important differences between countries and studies. These differences are partly due to differences in definitions, sampling strategies, and data handling methods. For example, the mean of a range is not always adequate to account for the large variations between regions [34, 35] and practices [36]. Also, many studies do not describe in detail the nature of the contacts investigated (face-to-face, telephone etc.). Furthermore, several studies do not differentiate between contacts with physicians and those with practice nurses or practice assistants, whereas others do [37, 38]. Still, the following cautious conclusions can be drawn from Table 4:

- There is a remarkable correspondence between the two internationally comparative studies on GP workload by patients per week and consultation lengths in minutes (rows 1 and 2) with regard to the ranking of the countries under study. This is the case for the data on Germany, the Netherlands, and the UK. In both studies, German GPs see more patients in shorter consultations, whereas GPs in the UK and the Netherlands see more than one third fewer patients. In the Commonwealth study (row 2), the GPs in the United States have the lowest number of patients per week, combined with relatively long consultations.
- Several studies on the number of physicians visited and the number of contacts with them in the USA (rows 3–7) suggest a number around 7 visits per year for the elderly, increasing up to 16 for highly multimorbid elderly. Especially in the case of multimorbidity, contacts with specialists seem to be more frequent than with primary care physicians. Even if this greater number of contacts with specialists compared to primary care physicians seems to be a feature unique to the USA, the absolute number of contacts – both with primary care physicians and specialists – and the number of contacted specialists are by no means striking when compared to other OECD countries (see below).
- Some other OECD countries show lower average numbers of contacts with physicians (e.g., the UK [rows 20–21] and Sweden [39]). Others show somewhat higher numbers (e.g., the Netherlands [rows 8–11], Switzerland [rows 17–19], Spain [rows 22–23], and Canada [rows 12–15]). In all studies, contact frequencies are substantially higher in cases of multimorbidity or especially when examining (elderly) high utilizers.
- When high utilization is examined, some studies find very high contact rates, such as 40 in Austria (row 27), 44 in Ontario (row 12), and 50 in British Columbia (row 14). In all of these cases, however, the sample consists of the 5–7 % of the highest utilizers. Considering the 19 % of the highest utilizers found in our study despite strict cutoffs (e.g., ≥ 50 contacts per year), many foreign studies use cutoffs for high utilization which could function as cutoffs for low use in German studies.

Even with all of these differences between the studies taken into account, the German figures far surpass those of most other industrialized countries. In this context, it should be kept in mind that the German figures do not include oral health services, most of the complementary medical services not covered by the health insurance, and the visits to emergency departments (currently estimated at 125/1,000 of the total population per year and rapidly increasing). Also, according to the study by Koch et al., no substantial difference between the type of contacts in German GP-practices in comparison to other countries are given, as German GPs declare that 70 % of their contacts with patients are personal contacts, a figure corresponding to a middle ranking (min: 66 %, max: 87 %) among the 11 countries in that study [40].

### Reasons

Further research is needed to explain these exceptional features of the German ambulatory healthcare system. Several reasons may contribute to this phenomenon, probably involving mechanisms on the side of the providers, the patients and the system itself. Among the reasons on the patients’ side, possibly greater attention to complaints and diseases, greater health related fears [41], tendencies to delegate responsibility from one self to physicians, and greater beliefs in procedural approaches to diagnosis may play a role. German physicians in ambulatory care may practice contact increasing strategies, e.g., by recall habits, client binding strategies, and competition with specialists. Also, the impact of bureaucratic directives of the insurance system on utilization (e.g., quarterly prescription regulations, remuneration specifics and budget limits, certification requirements for short time sick leave etc.) should be analyzed. Like in the United States, specialists in Germany play an important role in caring for common conditions, independent of the level of comorbidity and of the medical need for highly specialized care [3, 42]. This may be due to the particular “double specialist track” in Germany, a term describing the doubling of hospital based specialists working solely in the hospital (with an actual density of 1 hospital specialist [including resident specialists] to 814 inhabitants) by a supply of specialists working solely in ambulatory care (actual density of 1 specialist to 1,192 inhabitants) [43]. Furthermore, the German ambulatory healthcare system is based solely on the figure of the physician, whereas nurse practitioners and qualified registered nurses hardly play a role in the provision of services in the practices, which leads to a complete lack of alternatives to the physician in health services.

### Consequences

The high number of contacts in Germany is associated with smaller time slots per consultation when compared with the UK and especially with the USA (Table 4: [40, 42, 44, 45]). Other authors forwarded that in gatekeeping countries (with fixed lists of patients) GP consultations are longer, more time is spent talking with patients, especially about psychosocial aspects of their conditions, and the workload of general practitioners is lower [46]. Table 4, however, shows that this is not generally the case.

The main problem of high patient numbers per day and short consultation lengths may be that those patients who need intensive visits may not get them because the waiting room is always full, such as is the case in Germany, where it is not uncommon for GPs to see 70 patients per day. Østbye et al., who calculated the number of hours needed to provide guideline-conform care for the 10 most common chronic diseases in the USA, “provided the disease is stable and in good control”, found a requirement of 3.5 h a day. When adding uncontrolled disease, the estimated time required increased by a factor of 3. Applying this factor to all 10 diseases, time demands would increase to 10.6 h a day. The authors concluded that “current practice guidelines for only 10 chronic illnesses require more time than primary care physicians have available for patient care overall” [47]. German GPs are aware that their patient load does not allow them to allocate sufficient time per patient, and they consider a reduction of their patient load by around 25 % to be necessary for sufficient care [48].

The other danger of high utilization is that most of the contacts produce diagnoses, suggestions for further diagnostic procedures, referrals, drug prescriptions, physiotherapy prescriptions, and lifestyle recommendations that need to be understood by the patients and integrated into their lives. It is unclear how elderly patients handle this in their daily lives. Barbara Starfield concluded from several studies that “an excessive specialist supply with inappropriate specialist use leads to greater frequency of tests, more false-positive results, and worse outcomes than appropriate specialist use. The more physicians patients see, the greater the likelihood of adverse effects” [42]. As people’s use of specialist services is rapidly increasing, at least in the United States [42] and in Germany, the concepts of personal responsibility and self-management by (elderly) patients appear equally as fanciful as those stressing the need for continuity and comprehensiveness in medical care in these countries.

### Incentives for change?

In spite of the dissatisfaction on both sides – primary care physicians and patients – professional and/or political initiatives to reduce the utilization rates have not occurred, so far. Politicians might fear that the introduction of visible restrictions to utilization may result in criticism for a presumed cutting the welfare state. The size of high utilization described in this paper is generally considered to be a social achievement rather than a societal problem of medical care. The political and public disinterest in the issue of utilization frequency is also demonstrated by the fact that a reform of the reimbursement system in ambulatory care in 2008 went ahead with a discontinuation of the administrative documentation of utilization rates. Since that year, the responsible institutions – statutory health insurance companies, statutory physicians’ associations, and also the government – consciously renounce to document data on contact and referral rates for Germany.

### Masking the facts

The exceptional figures for Germany have not been noticed internationally because the OECD publishes mistakable data on the contact rates in German ambulatory care. Under the heading “number of contacts with physicians”, OECD does not report the number of contacts (actually estimated at 20 contacts per year [49]) but the number of contacted practices per patient and year (e.g., 8.2 in 2009). By means of this unusual – and misleading – definition of “contact”, explained only in a rather cryptic footnote, Germany occupies a prominent but still inconspicuous position in the international rank order of contact numbers [50].

### High use or overuse?

Of course, high utilization rates do not mean unjustified and/or dangerous overuse *per se*. Also, apart from figures on the general population and for the elderly as described in this paper, specific figures for the German working population are lacking. Also, evidence for the motives behind high utilization cannot be found in claims data. As there is no objective criterion for high utilization and, thus, no valid reference for diagnosing overuse, the very high utilization figures in German ambulatory medical care do not allow to use the term overutilization. On the other hand, the far-above-neighbor OECD countries utilization figures do not correspond to German outcome figures [51]. Therefore, questions arise about the relationship between benefit and harm of such high contact rates. As the utilization of inpatient care in Germany is also at the top of the OECD ranking list [18], more research is needed on the reasons and motivations for utilization rates in both healthcare sectors as well as on the interaction between the two [52]. In contrast, older people generally need a comprehensive and coordinated treatment plan, which is difficult to achieve with a two-digit number of contacts with specialists, especially considering the well-known deficits of communication between these specialists [53].

### Strengths and weaknesses

This study is based on claims data and therefore shows strengths and weaknesses inevitably related to this database. Apart from age and gender, no further socio-demographic variables were gathered that could be used to explain the differences in prevalence and risk. By including 46 diagnoses, we assume that all common and serious chronic diseases have been investigated. These diagnoses are documented by physicians – GPs and specialists – working in statutory healthcare and thus not by specially trained study personnel. It is known that there are limits to the validity of coded, claims related diagnoses [54, 55]. Validity issues of this nature were also found in the primary data collected via interviews with general practitioners [56, 57]. On the other hand, claims data allow a larger study population, including patients that are hard to reach in field studies, such as nursing home residents, very elderly patients, and severely ill patients. Memory bias regarding utilization and a bias due to socially acceptable answers of patients are excluded. This is the case for provider data as well. Finally, this cross-sectional study can detect associations but not causes.

The claims data stem from one insurance company, which poses the question of representativity. As there was and still is a high number of insurances (some 150 around 2006) some operating at regional and others on a national level, there is no possibility to draw a representative sample of the insured population in the strict sense of the term. The few studies comparing data from several insurers show that both problems of internal and external validity are given. With regard to internal validity, the GEK-Data show a higher proportion of males for historical reasons. As analyzed in a previous study, these gender difference does not have a major impact on utilization figures in the elderly population insured by the GEK [3]. As for external validity, comparisons between insurances are limited to the epidemiology of certain diseases, and studies on utilization data are lacking [58, 59]. Exercising extreme caution we may postulate from other data sources on insurance characteristics that there is no reason to assume that data from GEK-sources are exceptional or extreme.

## Conclusions

Germany is likely to be at the top of the rank order of utilization of ambualtory healthcare (at least) among the elderly. Followers are far behind. Reamarkably, a discussion of this phenomenom is absent in Germany.

The high utilization of ambulatory care physicians appears to be a multilayered, complex phenomenon based on many chronic diseases of varying origin. Both serious somatic illnesses and psychological complaints lead to high utilization. Also, health system characteristics and incentives from providers might play an important role. Complex problems require complex solutions. Therefore, an optimization of the utilization of statutory healthcare requires a multidimensional approach that goes beyond financial restrictions alone. Further research is needed both regarding the reasons and the remedies of this very high utilization of ambulatory medical care by the elderly in Germany and, perhaps, also by the working-age population.

The comparison of studies on utilization of physicians suffer from imprecision of definitions of essential terms like contact, visit, consultation etc. This is also the case for consultation length and referral rate. These imprecisions make international comparisons difficult. Hence, OECD – and other insitutions – should point more explicitely at this problem in their publications and untertake steps towards harmonization.

### Data availibilty

The data of this study are property of the BARMER GEK Krankenversicherung sited at Wuppertal, Germay. Access to the data will need an application at the insurance headquarter.
